# Refractory circulatory failure in COVID-19 patients treated with veno-arterial ECMO a retrospective single-center experience

**DOI:** 10.1371/journal.pone.0298342

**Published:** 2024-04-01

**Authors:** Clemens Wiest, Alois Philipp, Maik Foltan, Florian Geismann, Roland Schneckenpointer, Simon Baumgartner, Florian Sticht, Florian Hitzenbichler, Michael Arzt, Christoph Fisser, Andrea Stadlbauer, Thomas Dienemann, Lars Siegfried Maier, Dirk Lunz, Thomas Mueller, Matthias Lubnow

**Affiliations:** 1 Department for Internal Medicine II, University Hospital Regensburg, Regensburg, Germany; 2 Department of Cardiothoracic Surgery, University Hospital Regensburg, Regensburg, Germany; 3 Center for Pneumonology, Donaustauf Hospital, Donaustauf, Germany; 4 Department of Infection Prevention and Infectious Diseases, University Hospital Regensburg, Regensburg, Germany; 5 Department of Surgery, University Hospital Regensburg, Regensburg, Germany; 6 Department of Anesthesiology, University Hospital Regensburg, Regensburg, Germany; The Open University, UNITED KINGDOM

## Abstract

**Objective:**

In this retrospective case series, survival rates in different indications for veno-arterial extracorporeal membrane oxygenation (VA-ECMO) and differential diagnoses of COVID-19 associated refractory circulatory failure are investigated.

**Methods:**

Retrospective analysis of 28 consecutive COVID-19 patients requiring VA-ECMO. All VA-ECMO’s were cannulated peripherally, using a femoro-femoral cannulation.

**Results:**

At VA-ECMO initiation, median age was 57 years (IQR: 51–62), SOFA score 16 (IQR: 13–17) and norepinephrine dosing 0.53μg/kg/min (IQR: 0.35–0.87). Virus-variants were: 61% wild-type, 14% Alpha, 18% Delta and 7% Omicron. Indications for VA-ECMO support were pulmonary embolism (PE) (n = 5, survival 80%), right heart failure due to secondary pulmonary hypertension (n = 5, survival 20%), cardiac arrest (n = 4, survival 25%), acute heart failure (AHF) (n = 10, survival 40%) and refractory vasoplegia (n = 4, survival 0%). Among the patients with AHF, 4 patients suffered from COVID-19 associated heart failure (CovHF) (survival 100%) and 6 patients from sepsis associated heart failure (SHF) (survival 0%). Main Complications were acute kidney injury (AKI) 93%, renal replacement therapy was needed in 79%, intracranial hemorrhage occurred in 18%. Overall survival to hospital discharge was 39%.

**Conclusion:**

Survival on VA-ECMO in COVID-19 depends on VA-ECMO indication, which should be considered in further studies and clinical decision making. A subgroup of patients suffers from acute heart failure due to inflammation, which has to be differentiated into septic or COVID-19 associated. Novel biomarkers are required to ensure reliable differentiation between these entities; a candidate might be soluble interleukin 2 receptor.

## Introduction

### Objective

More than 6 million people died because of COVID-19 worldwide [1]. According to epidemiological calculations, between January 2020 and December 2021, 18.2 million people died within the context of the SARS-CoV2 pandemic [2]. Thousands of patients needed veno-venous extracorporeal membrane oxygenation (VV-ECMO) [3], the mortality varied, ranging from 23% to 73%. Besides pulmonary support, circulatory support by veno-arterial extracorporeal membrane oxygenation (VA-ECMO) was necessary in COVID-19 patients [4] with high mortality rates up to 72% [5]. Common indications for circulatory support by VA-ECMO are pulmonary embolism (PE) or circulatory failure due to myocardial infarction or myocarditis [6]. Beside these usual indications, patients needed circulatory support due to COVID-19 specific entities, such as COVID-19 induced myocarditis [7] or COVID-19 associated heart failure (CovHF). However, treatment of refractory circulatory failure due to COVID-19 specific entities is challenging, especially because only very little evidence on indications, outcomes and differential diagnoses of underlying pathology is available. This retrospective study evaluates indications for VA-ECMO and differential diagnoses in COVID-19 associated refractory circulatory failure.

## Methods

Retrospective analysis of all consecutive ICU-admitted patients at the University hospital Regensburg (UKR) with PCR proven COVID-19 infection (n = 28) between March 2020 and March 2022, requiring VA-ECMO.

At our institution the criteria for VA-ECMO in patients with COVID-19 were consistent with the current ESLO criteria and recommendations. Additionally, the decision for or against ECMO war always taken from at least two experienced intensivists. Regarding time of ventilation before ECMO our center allowed up to two weeks of ventilation prior ECMO. The ECPR program was maintained during the pandemic.

All included patients were cannulated for VA-ECMO peripherally (femoro-femoral cannulation).

ECMO-Weaning in VA-ECMO has been done under echocardiographic guidance as follows, blood flow was reduced in steps of 300ml-500ml per day, when the patient was hemodynamically stable (low levels of vasoactives: Dobutamin <20mg/h, norepinephrin < 0,5mg/h, epinephrin < 0,1mg/h), normal lactate, central venous saturation >65%, and no other signs of organ malperfusion. If blood flow is 1.5 l/min or less (we do not reduce blood flow below 1.2 l/min), there is no sign of impending infection, no severe hypervolemia and the patient is clinically stable on no/low dose vasopressors, we usually remove VA-ECMO. Usually before decannulation a back flow test is performed. During this test the blood pump is stopped with back flow of blood from the arterial to the venous site (about 400ml/min). If the patients MAP does not decrease significantly, the removal of the ECMO was scheduled. The removal of the arterial cannulas was done with closure devices (Manta ^®^ closure device), surgically (if implantation was done surgically, ischemic complications were present or in case of severe obesity) or by manual compression. The removal of the venous drainage cannulas was done by manual compression and a skin/subcutaneous suture [8].

### Definitions

Survival was defined as survival to hospital discharge from UKR. KDIGO criteria defined acute kidney failure.

At VA-ECMO initiation, patients with acute heart failure (AHF) were considered to suffer from sepsis associated heart failure (SHF) or COVID-19 associated heart failure (CovHF) considering laboratory findings (lower CRP and higher IL6 levels in CovHF) and clinical presentation (acute heart failure without signs of severe sepsis in CovHF). Further inflammatory markers (interleukin 8, sIL2-receptor and TNF) were not determined on a daily basis and were therefore not available for therapy decision.

Patients with right heart failure (RHF) were differentiated in those with PE and those without evidence of PE in CT scan (secondary RHF).

Vasoplegia was considered if ventricular systolic function was normal or unchanged compared to previous examinations combined with high doses of catecholamines and defined according to the criteria used in [9].

Daily life performance was measured with WHO performance status, ranging from 1 (normal daily activity) to 4 (completely dependent on nursing assistance).

Neurological outcome was measured with cerebral performance category (CPC) ranging from 1 (normal) to 5 (brain death).

### Data acquisition

We used the ECMO registry of the University Hospital Regensburg (UKR) and data of the electronical patient chart for this retrospective analysis (patient-related data, ECMO characteristics, ventilator settings, medication, hemodynamic data, laboratory values and complications). The database was accessed on 1^st^ February 2023, authors could not identify individual patients. The requirement of individual patient consent and necessity of approval for the data report was waived by the University of Regensburg ethics committee (ethic approval number; 22-2898-104, April 04, 2022.)

### Statistical analysis

Statistical analysis was performed with SPSS (IBM, New York USA) version 26.0. Descriptive statistics are presented with number and percentage for categorical variables and with median (25th–75th percentile interquartile range (IQR)) for continuous variables. Between-group comparisons of categorical and metric variables were performed using Fisher’s exact test or Chi-square test of independence as indicated or the Mann-Whitney U-test. P values below 0.05 were considered statistically significant.

## Results

### Patients

**Fig 1:**
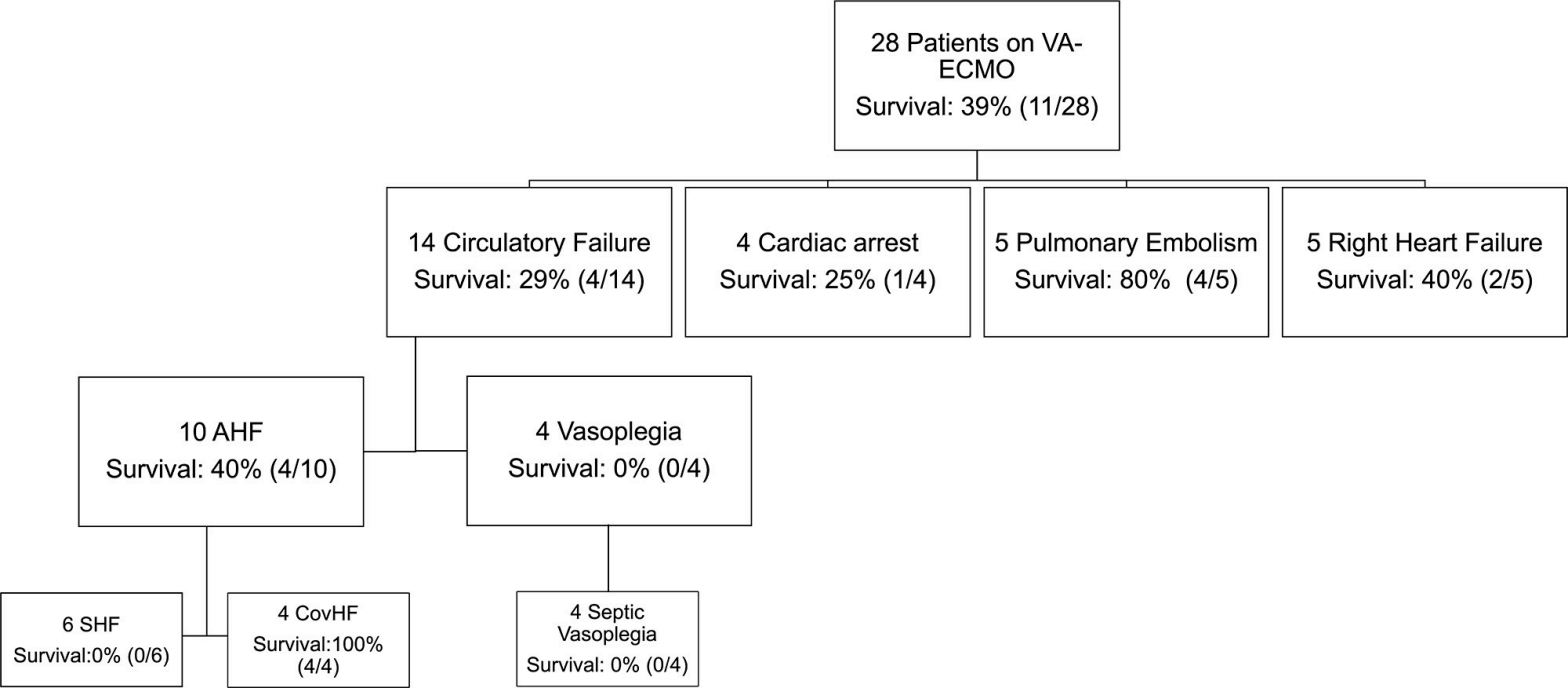
Indications for VA-ECMO among patients with COVID-19 and indication specific survival. AHF: Acute heart failure, SHF: Sepsis associated heart failure. CovHF: COVID-19 associated heart failure. All 4 patients with septic vasoplegia had sepsis due to mycotic infection.

28 Patients underwent peripheral VA-ECMO therapy. At VA-ECMO initiation, median age was 57 years (IQR: 51–62), Sequential organ failure assessment (SOFA) score 16 (IQR: 13–17) and norepinephrine dosing 0.53μg/kg/min (IQR: 0.32–0.78). Virus-variants were: 61% wild-type, 14% Alpha, 18% Delta and 7% Omicron. Indications for VA-ECMO support were pulmonary embolism (PE) (n = 5), right heart failure due to secondary pulmonary hypertension (without PE) (n = 5), cardiac arrest (n = 4), acute heart failure (AHF) (n = 10) and refractory vasoplegia (n = 4). Secondary right heart failure occurred 6 (3–13) days after first symptoms of COVID infection. Characteristics of patients with right heart failure are summarized in S6 Table. Reasons for cardiac arrest and resulting ECPR were: rhythmogenic (n = 3) and pericardial effusion (n = 1) (S4 Table). Overall survival to hospital discharge was 39% (11/28) and depended on indication for VA-ECMO (Fig 1). 17 patients were merely supported with VA-ECMO (survival 41%), 3 patients were switched from VV-ECMO to VA-ECMO (survival 0%), and 8 patients were converted from VA to veno-arterial-venous (VAV) or VV-ECMO (survival 50%) (S1 Fig). Switch in configuration was needed in median after 4 (3–6) days after initiation of first ECMO mode. ECMO configurations and related clinical complications are summarized in S5 Table. Causes of death are summarized in S3 Table.

In those patients with pulmonary embolism (PE), cardiac arrest (CA) or isolated right heart failure (RHF) underlying pathology causing circulatory failure was evident. In contrast, in the 14 other patients presenting with circulatory failure, underlying disease was not clear. These patients presented with higher inflammatory markers before VA-ECMO (Table 1). All patients with acute heart failure (AHF) received Levosimendan therapy, other vasoactive medication is summarized in Table 1 and S1 Table.

**Table 1:** Baseline characteristics. Continuous variables are shown as median and IQR 25^th^- 75^th^. Categorized variables are shown as number and percentage of group’s size. Statistical differences between groups were calculated using Whitney-Man-U Test for continuous variables and Chi-Square test for categorized variables. Differences in COVID-Treatment were not calculated due to small case number. BMI: Body mass index, SOFA: sequential organ failure assessment, ECMO: extracorporeal membrane oxygenation, PEEP: positive end-exspiratory pressure, TV: Tidal-volume. ASAT: Aspartataminotransferase, ALAT: Alaninaminotransferase, LDH: Lactatdehydrogenase. P/F Ratio: PaO2 / FiO2 ratio = Horrowitz Index.

	All (n = 28)	PE/CA/RHF	Circulatory failure	p-value
		n = 14	n = 14	
Age	57.3 (51.4–61.8)	57.4 (53.5–62.5)	57.1 (46.9–61.8)	0.51
Male Sex	20 (71)	10 (71)	10 (71)	1
BMI (kg/m^2^)	29.4 (26.8–33.1)	28.0 (25.7–32.8)	30.6 (27.6–35.5)	0.10
Primary on VA-ECMO	25 (89)	13 (93)	12 (86)	
Primary on VV-ECMO	3 (11)	1 (7)	2 (14)	
Days from First Symptoms to intubation	2 (1–6)	4 (1–15)	2 (1–4)	0.51
Days from First Symptoms to ECMO	8 (2–19)	6 (2–19)	11 (3–18)	0.51
SOFA	16 (13–17)	14 (13–19)	16 (13–17)	0.88
ECMO Duration (days)	8 (4–16)	9 (3–22)	7 (5–9)	0.45
**Pre-Existing Disease**				
Arterial Hypertension	15 (54)	8 (57)	7 (50)	0.71
Diabetes mellitus	7 (25)	1 (7)	6 (43)	**0.03**
Chronic Kidney Insufficiency	7 (25)	2 (14)	5 (26)	0.19
Immunosuppression	2 (7)	1 (7)	1 (7)	1
Vascular Disease	3 (11)	1 (7)	2 (14)	0.54
Cardiac Disease	3 (11)	2 (14)	1 (7)	0.54
Solid Organ Transplantation	2 (7)	1 (7)	1 (7)	1
**Virus Variant**				0.63
Wildtype	17 (61)	7 (50)	10 (71)	
Alpha-Variant	4 (14)	3 (21)	1 (7)	
Delta-Variant	5 (18)	3 (21)	2 (14)	
Omikron	2 (7)	1 (7)	1 (7)	
**COVID-19 Specific Treatment**				
Glucocorticoids	25 (89)	12 (86)	13 (93)	
Monoclonal Antibodies	3 (11)	1 (7)	2 (14)	
Reconvalescent Plasma	12 (42)	5 (26)	7 (50)	
Remdesivir	7 (25)	3 (21)	4 (29)	
Tocilizumab	1 (4)	0 (0)	1 (7)	
Anakinra	4 (14)	0 (0)	4 (29)	
Plasmapheresis	9 (32)	0 (0)	9 (64)	
**Laboratory Testing at ECMO Initiation**				
White blood cells (/nl)	16.6 (9.3–23.9)	16.6 (7.5–25.9)	18.1 (9.3–23.2)	0.83
Lymphocytes (/nl)	1.3 (0.7–1.7)	1.3 (0.6–1.8)	1.3 (0.8–1.7)	0.44
Platelets (/nl)	241 (102–288)	200 (126–337)	225 (88–287)	0.72
Creatinine (mg/dl)	2.1 (1.1–3.0)	2.0 (1.2–2.7)	2.2 (1.1–3.2)	1.0
Blood Urea (mg/dl)	76 (42–115)	61 (39–103)	83 (45–119)	0.33
Bilirubine (mg/dl)	1.0 (0.5–2.4)	1.0 (0.4–1.8)	1.4 (0.8–4.4)	0.24
ASAT (U/L)	218 (80–837)	142 (87–3302)	227 (79–633)	0.52
ALAT (U/L)	131 (66–634)	121 (70–1709)	148 (47–221)	0.35
LDH (U/L)	809 (459–2839)	804 (531–2582)	900 (430–1614)	0.83
CRP (mg/L)	187 (60––294)	139 (38–2001)	247 (162–379)	**0.03**
Procalcitonin (ng/ml)	4 (1.1–11.3)	4.7 (2.7–23.0)	2.0 (0.9–7.9)	0.17
Ferritin (ng/ml)	6142 (2806–30944)	5555 (1424–31535)	6142 (4108–31904)	0.91
IL-6 (pg/ml)	455 (115–1249)	144 (90–516)	1083 (391–2234)	**0.006**
IL-8 (ng/L)	188 (81–560)	94 (67–153)	560 (334–1143)	**<0.001**
TNF (pg/ml)	21.5 (14.0–31.2)	15.0 (10.5–21.0)	31.1 (22.5–44.5)	**0.001**
s-IL2-R (U/ml)	2013 (869–3482)	2063 (936–3615)	1964 (831–3390)	1
**Hemodynamics at VA-ECMO Initiation**				
Mean Arterial Pressure (mmHg)	65 (55–68)	61 (51–67)	65 (55–84)	0.15
Norepinephrine (μg/kg/min)	0.53 (0.35–0.87)	0.53 (0.04–0.73)	0.70 (0.43–1.01)	0.16
	N = 25	N = 11	N = 14	
Epinephrine (μg/kg/min)	0.0 (0.0–0.08)	0.0 (0.0–0.08)	0.01 (0.0–0.22)	0.30
	N = 12	N = 5	N = 7	
Vasopressin (IE/h)	0.52 (0.0–2)	0.0 (0.0–1)	1.0 (0.04–2.0)	**0.02**
	N = 15	N = 4	N = 11	
Dobutamine (mg/h)	0.0 (0.0–10)	0.0 (0.0–10)	10 (0–11)	0.31
	N = 13	N = 5	N = 8	
Lactate (mg/dl)	56 (24–95)	56 (19–100)	55 (33–96)	0.58
**Ventilator Settings and Blood Gas Analysis at VA-ECMO Initiation**				
PEEP (mbar)	13 (12–15)	12 (12–16)	14 (12–15)	0.59
Pmax (mbar)	30 (26–36)	31 (27–34)	30 (25–37)	0.67
Driving Pressure (mbar)	15 (14–21)	17 (14–22)	16 (14–21)	0.62
TV (ml)	543 (450–637)	452 (413–580)	565 (491–642)	0.09
TV (ml/kg PDBW)	7.5 (6.3–8.9)	6.7 (5.4–8.1)	8.1 (6.8–9.2)	0.07
pH	7.17 (7.1–7.21)	7.19 (7.02–7.32)	7.16 (7.10–7.21)	0.67
P/F Ratio	71 (59–156)	71 (58–108)	81 (59–196)	0.60
paCO_2_ (mmHg)	53 (42–65)	55 (41–66)	52 (42–66)	0.83
**Complications during ECMO Therapy**				
Acute Kidney Injury	26 (93)	12 (86)	14 (100)	0.14
Renal Replacement Therapy	22 (79)	10 (71)	12 (71)	0.36
Intracranial hemorrhage	5 (18)	2 (7)	3 (21)	0.25
Ischemic Stroke	1 (4)	0 (0)	1 (7)	0.19
Pulmonary Embolism	10 (36)	5 (36)	5 (36)	0.87

Ten of these patients with circulatory failure (Fig 1) presented with new onset severely reduced biventricular systolic function (AHF), in nine of them obstructive coronary artery disease was excluded. In six patients with AHF (Fig 1), myocarditis could be excluded (3 in myocardial biopsy, 3 in autopsy). Four patients with circulatory failure and no previous history of immunosuppression had invasive fungal infections (2 Aspergillosis, 1 invasive candidiasis, 1 Mucormycosis), all of them presented with vasoplegia and all of them died (Fig 1). 3 of these infections were identified in autopsy only. In 6 patients with AHF there was evidence of non-invasive aspergillus infection two days before VA-ECMO initiation. However, these infections were non-invasive and were not thought to be causative for circulatory failure.

Among the patients with AHF, 4 patients suffered from COVID-19 associated heart failure (CovHF) (survival 4/4) and 6 patients to sepsis associated heart failure (SHF) (survival 0/6).

**Fig 2:**
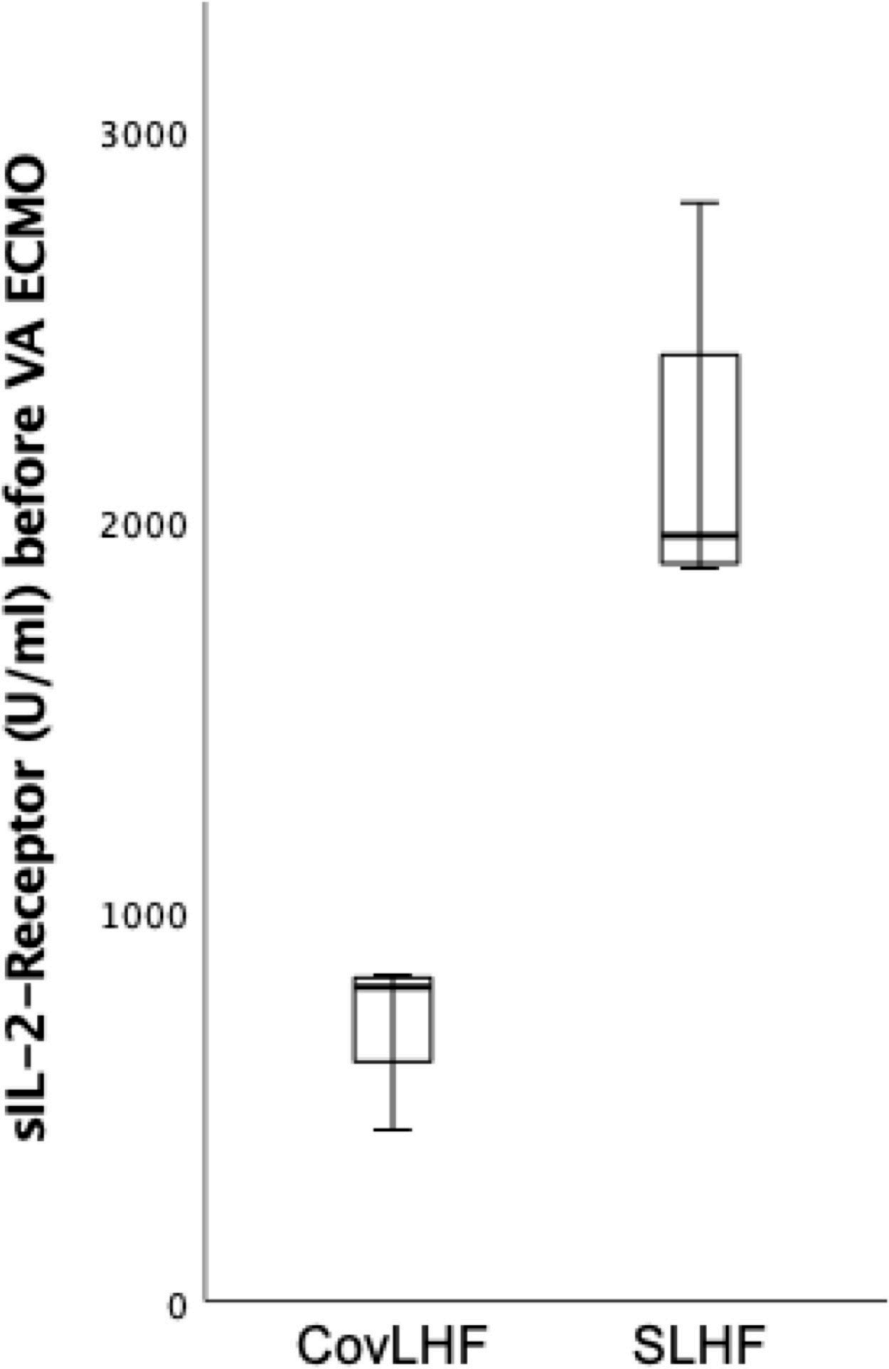
Soluble interleukin 2 receptor (sIL2-R) before VA-ECMO in COVID-19 associated heart failure and septic patients. CovHF: COVID-19 associated heart failure, SHF: Sepsis associated heart failure.

In patients with SHF, soluble Interleukin 2 receptor (sIL2-R) was higher compared to patients with CovHF (Fig 2, Table 2).

**Table 2:** Inflammatory and echocardiographic characteristics and specific treatments in comparison between patients with unclear circulatory failure, dichotomized in either probable CovHF or SHF. Variables are shown as median and IQR 25^th^–75^th^. Statistical difference between groups was calculated using Whitney-Man-U Test. SHF: Sepsis associated heart failure. CovHF: COVID-19 associated heart failure. CRP: C-reactive protein, PCT: Procalcitonin, TNF: Tumor-Necrosis Factor, H-Score for reactive hemophagocytic syndrome estimates the risk for having reactive hemophagocytic syndrome.

	CovHF, n = 4	SHF, n = 6	p-value
Survival	4 (100)	0 (0)	0.39
CRP (mg/dl)	219 (53–546)	255 (237–379)	0.56
PCT (ng/ml)	2.0 (0.7–3.5)	1.3 (0.7–7.4)	1
Ferritin (ng/ml)	11815 (5861–72534)	54508 (6490–103688)	0.48
Interleukin 6 (pg/ml)	953 (158–2598)	991 (428–5496)	0.61
Interleukin 8 (ng/L)	458 (152–549)	624 (237–1143)	0.34
sIL2-R (U/ml)	807 (527–834)	1964 (1887–2623)	**0.016**
TNF (pg/ml)	22.5 (15.5–42.3)	31.1 (21.0–42.0)	0.56
H-Score	99 (73–136)	122 (68–159)	0.61
**Specific Treatment**			
Glucocorticoids	4 (100)	6 (100)	
Monoclonal Antibodies	2 (50)	0 (0)	
Reconvalescent Plasma	2 (50)	3 (50)	
Remdesivir	1 (25)	2 (33)	
Tocilizumab	0 (0)	0 (0)	
Anakinra	2 (50)	1 (17)	
Plasmapheresis	4 (100)	4 (67)	

All 4 patients with CovHF (survival 4/4) and 4 patients with SHF (survival 0/4) were treated with plasmapheresis. Specific treatments in patients with circulatory failure are summarized in Table 1 and shown per patient in S2 Table.

In all patients, independent from ECMO configuration or underlying pathology, the rate of clinical complications was high (Table 1).

Only one patient died 8 days after referral to another hospital. All survivors have WHO performance status 1. All except for one patient have cerebral performance category 1.

## Discussion

Data about survival of COVID-19 patients on VA-ECMO is limited [10]. Only few studies report survival rates of patients on VA-ECMO due to COVID-19 ranging from 27.9% to 36% [4, 5, 11]. In our study, overall survival was 39% which is line with the available data, especially data from the EuroELSO registry [12, 13]. These studies found also low survival rates in hybrid ECMO configurations [12, 13]. The mentioned studies evaluated survival in COVID-19 patients independent from underlying pathology causing circulatory insufficiency. A case series of 9 patients suffering from COVID-19 associated myocarditis found a survival of 77.8% [7]. In our study, survival rates are varying, dependent on underlying disease, causing circulatory failure (Fig 1). Another important factor for survival is patient selection. Our selection criteria were consistent with the published criteria [14], except for 4 patients with COVID-19 and ECPR, which was discussed as contraindication for VA-ECMO in interim guidelines [14]; in 3 patients, receiving ECPR out of hospital, COVID-19 infection was not known. One patient was resuscitated in hospital due to pericardial effusion during cannulation for awake VV-ECMO as rescue measure.

The best survival in our case series was seen in patients with pulmonary embolism (80%) and patients with AHF due to CovHF (100%). This points out the major importance of underlying pathology and its treatment. The rate of complications, especially kidney failure and need of renal replacement therapy among all patients was high, even compared to literature (70%) and shows the complexity and disease severity of this COVID-19 patient group [5, 13].

In our study, patients with AHF or vasoplegia presented with higher inflammatory markers compared to the other patients, indicating an underlying inflammatory disease in these patients. In COVID-19 patients, severe inflammation, the so called “cytokine storm”, was described as reason for COVID-19 associated circulatory failure [15]. In these patients anti-cytokine therapy and immunosuppression were found to be beneficial [16]. In our study, 4/10 patients with AHF were thought to have acute cardiac failure due to COVID-19 (CovHF). Because of imperative treatment consequences, it is necessary to determine if the underlying disease is more likely infectious or inflammatory including a detailed microbiological workup. Among those patients with AHF, sIL2-R was significantly higher in patients with SHF compared to these patients with CovHF (Fig 2, Table 2). Previous studies found an association between levels of sIL2-R and sepsis severity [17] and higher levels of sIL2-R in sepsis compared to aseptic inflammation [18] and higher levels of sIL2-R in bacteremic sepsis compared to non-bacteremic systemic inflammation [19]. These experimental findings support our clinical observation, therefore higher sIL2-R levels might help to differentiate between sepsis associated heart failure and non-septic heart failure, in our cohort COVID-19 associated heart failure. However, further studies are needed to confirm our results and give better understanding of cytokine profiles in different clinical pathologies.

We found plasmapheresis was helpful to improve patient’s hemodynamics in patients with circulatory failure and inflammation. Plasmapheresis allowed time to further characterize the underlying disease without primarily initiating immunosuppression.

All 6 patients with SHF died, which contrasts with the current literature [20, 21]. The reasons are speculative but could be caused by more invasive fungal infections, the administration of steroids in most of these patients and an immunosuppression caused by SARSCoV2 infection itself.

All patients (n = 4) with circulatory failure, preserved left ventricular function and vasoplegia died, which is in line with data from a large meta-analysis from patients on VA-ECMO due to septic shock, describing poor survival in patients with preserved left ventricular function [21].

In this study by Ling R et al. a pooled survival in those with LV-EF >35% of 32% was described, which is only half of the survival of those with LV-EF <20% (62%), but not futile. Therefore, in very selected patients, especially younger ones, we offered VA-ECMO to patients with vasoplegia, but failed to gain a survival benefit. In our case series these 4 patients were found to have invasive fungal infections. In 3 of them, fungal infection was unrecognized and found in autopsy only. Therefore, a detailed microbiological workup is necessary in these patients. Unfortunately, 3 of these patients had been on appropriate antifungal therapy which couldn’t improve the clinical course.

### Limitations

The main limitations of our study are the small number of cases and the single center retrospective study design, which limits the generalizability of the shown data. Prospective studies are needed to evaluate the value of our results.

## Conclusion

Survival of patients on VA-ECMO and COVID-19 depends on underlying pathology. This should be considered in further studies and clinical decision making. A subgroup of patients suffers from acute heart failure due to inflammation, which has to be differentiated into septic or COVID-19 associated due to different therapeutic approaches. Novel biomarkers are required to ensure reliable differentiation between these entities; a candidate might be soluble interleukin 2 receptor.

## Supporting information

S1 Fig ECMO configurations and configuration-conversion. ECMO configuration and ECMO configuration-conversions among patients with COVID-19 and configuration specific survival. OHCA: out of hospital cardiac arrest. VA: veno-arterial, VAV: veno-arterial-venous, VV: veno-venous.
(DOCX)

S1 Table Baseline characteristics of survivors and non-survivors of VA-ECMO therapy. Continuous variables are shown as median and IQR 25^th^- 75^th^. Categorized variables are shown as number and percentage of group’s size. Statistical difference between groups was calculated using Whitney-Man-U Test for continuous variables and Chi-Square test for categorized variables. Differences in COVID-Treatment and were not calculated due to small case number. *n = 12, **n = 4, ***n = 8.
(DOCX)

S2 Table Patients with circulatory failure and their specific treatment. Summarizes the Patients with Circulatory failure and their specific treatment. * This patient had preexisting reduced LV impairment without deterioration before ECMO.
(DOCX)

S3 Table Causes of death dependent on ECMO indication. Summarizes the causes of death dependent of ECMO indication. ICH: Intracranial hemorrhage.
(DOCX)

S4 Table Patients with cardiac arrest. Characterizes patients with cardiac arrest. VF: ventricular fibrillation, PEA: pulseless electric activity. NSE: Neuron specific Enolase.
(DOCX)

S5 Table ECMO configurations and related complications. Summarizes ECMO configurations and related complications. CFH: cell free Hemoglobin.
(DOCX)

S6 Table Characteristics of patients with right heart failure. Characterizes patients with right heart failure (RHF). Continuous variables are shown as median and IQR 25^th^- 75^th^. Categorized variables are shown as number and percentage of group’s size.
(DOCX)
