# Supplementary material for: Refractory circulatory failure in COVID-19 patients treated with veno-arterial ECMO a retrospective single-center experience
Source: PLoS One. 2024 Apr 1;19(4):e0298342. doi: 10.1371/journal.pone.0298342 (PMC10984404; doi:10.1371/journal.pone.0298342)
Supplement: S1 Fig — ECMO configuration and ECMO configuration-conversions among patients with COVID-19 and configuration specific survival. OHCA: out of hospital cardiac arrest. VA: veno-arterial, VAV: veno-arterial-venous, VV: veno-venous. (DOCX) [file pone.0298342.s001.docx]

***Figure S1: ECMO Configurations and Configuration-conversion.***

*ECMO configuration and ECMO configuration-conversions among patients with COVID-19 and configuration specific survival. OHCA: out of hospital cardiac arrest. VA: veno-arterial, VAV: veno-arterial-venous, VV: veno-venous.*
