# Supplementary material for: Refractory circulatory failure in COVID-19 patients treated with veno-arterial ECMO a retrospective single-center experience
Source: PLoS One. 2024 Apr 1;19(4):e0298342. doi: 10.1371/journal.pone.0298342 (PMC10984404; doi:10.1371/journal.pone.0298342)
Supplement: S1 Table — Continuous variables are shown as median and IQR 25th- 75th. Categorized variables are shown as number and percentage of group’s size. Statistical difference between groups was calculated using Whitney-Man-U Test for continuous variables and Chi-Square test for categorized variables. Differences in COVID-Treatment and were not calculated due to small case number. *n = 12, **n = 4, ***n = 8. (DOCX) [file pone.0298342.s002.docx]

**Table S1: Baseline characteristics of Survivors and Non-Survivors of VA-ECMO therapy.**

|  | **All (n=28)** | **Survivor (n=11)** | **Non-Survivor (n=17)** | **p-value** |
| --- | --- | --- | --- | --- |
| Age | 57.3 (51.4 – 61.8) | 54.6 (49.9 – 59.9) | 57.8 (51.7 – 62.8) | 0.29 |
| Male Sex | 20 (71) | 8 (73) | 12 (71) | 0.90 |
| BMI (kg/m^2^) | 29.4 (26.8 – 33.1) | 28.2 (25.7 – 33.2) | 29.9 (27.4 – 33.5) | 0.31 |
| Primary on VA-ECMO | 25 (89) | 11 (100) | 14 (82) |  |
| Primary on VV-ECMO | 3 (11) | 0 (0) | 3 (18) |  |
| Days from First Symptoms to intubation | 2 (1 – 6) | 2 (1 – 13) | 2 (1 – 5) | 0.55 |
| Days from First Symptoms to ECMO | 8 (2 – 19) | 6 (3 – 29) | 9 (1 – 18) | 0.82 |
| SOFA | 16 (13 – 17) | 13 (12 – 17) | 17 (14 – 17) | 0.06 |
| ECMO Duration (days) | 8 (4 – 16) | 8 (3 – 21) | 7 (5 – 12) | 0.61 |
| **Pre-Existing Disease** | | | | |
| Arterial Hypertension | 15 (54) | 2 (18) | 13 (77) | **0.003** |
| Diabetes mellitus | 7 (25) | 3 (27) | 4 (24) | 0.82 |
| Chronic Kidney Insufficiency | 7 (25) | 2 (18) | 5 (29) | 0.50 |
| Immunosuppression | 2 (7) | 0 (0) | 2 (12) | 0.24 |
| Vascular Disease | 3 (11) | 1 (9) | 2 (12) | 0.82 |
| Cardiac Disease | 3 (11) | 1 (9) | 2 (12) | 0.82 |
| Solid Organ Transplantation | 2 (7) | 0 (0) | 2 (12) | 0.24 |
| **Virus Variant** | | | | 0.94 |
| Wildtype | 17 (61) | 6 (55) | 11 (66) |  |
| Alpha-Variant | 4 (14) | 2 (18) | 2 (12) |  |
| Delta-Variant | 5 (18) | 2 (18) | 3 (18) |  |
| Omikron | 2 (7) | 1 (9) | 1 (6) |  |
| **COVID-19 Specific Treatment** | | | | |
| Glucocorticoids | 25 (89) | 10 (91) | 15 (88) |  |
| Monoclonal Antibodies | 3 (11) | 2 (18) | 1 (6) |  |
| Reconvalescent Plasma | 12 (42) | 5 (30) | 7 (41) |  |
| Remdesivir | 7 (25) | 3 (27) | 4 (24) |  |
| Tocilizumab | 1 (4) | 0 (0) | 1 (6) |  |
| **Laboratory Testing at VA-ECMO Initiation** | | | | |
| White blood cells (/nl) | 16.6 (9.3 – 23.9) | 16.0 (8.9 – 22.9) | 18.6 (10.1 – 26.4) | 0.48 |
| Lymphocytes (/nl) | 1.3 (0.7 – 1.7) | 1.2 (0.6 – 1.4) | 1.4 (0.7 – 1.7) | 0.48 |
| Platelets (/nl) | 241 (102 – 288) | 235 (174 – 371) | 133 (90 – 283) | 0.18 |
| Creatinine (mg/dl) | 2.1 (1.1 – 3.0) | 1.7 (1.1 – 2.4) | 2.2 (1.3 – 3.8) | 0.48 |
| Blood Urea (mg/dl) | 76 (42 – 115) | 78 (42 – 114) | 73 (40 – 156) | 0.90 |
| Bilirubine (mg/dl) | 1.0 (0.5 – 2.4) | 0.8 (0.4 – 1.6) | 1.5 (0.6 – 5.5) | 0.11 |
| ASAT (U/L) | 218 (80 – 837) | 142 (80 – 865) | 270 (81 – 654) | 0.87 |
| ALAT (U/L) | 131 (66 – 634) | 121 (66 – 634) | 178 (64 – 716) | 0.90 |
| LDH (U/L) | 809 (459 – 2839) | 804 (600 – 1955) | 900 (268 – 1754) | 0.79 |
| CRP (mg/L) | 187 (60 – 294) | 183 (66 – 277) | 218 (35 – 355) | 0.88 |
| Procalcitonin (ng/ml) | 4.0 (1.1 – 11.3) | 2.9 (1.8 – 5.3) | 5.7 (0.9 – 23.0) | 0.40 |
| Ferritin (ng/ml) | 6142 (2806 – 30944) | 5623 (1113 – 29712) | 7503 (3409 – 31715) | 0.38 |
| IL-6 (pg/ml) | 455 (115 – 1249) | 198 (84 – 880) | 727 (146 – 1407) | 0.20 |
| IL-8 (ng/L) | 188 (81 – 560) | 94 (52 – 452) | 247 (110 – 850) | 0.06 |
| TNF (pg/ml) | 21.5 (14.0 – 31.2) | 19.0 (14.0 – 25.0) | 27.0 (14.0 – 39.0) | 0.38 |
| s-IL2-R (U/ml) | 2013 (869 – 3482) | 1792 (789 – 3165) | 2428 (1489 – 3752) | 0.16 |
| **Hemodynamics at VA-ECMO Initiation** | | | | |
| MAP (mmHg) | 65 (55 – 68) | 61 (55 – 66) | 65 (55 – 71) | 0.38 |
| Norepinephrine (µg/kg/min) | - 1. (0.32 – 0.78)   N=25 | 0.44 (0.09 – 0.71)  n=10 | 0.56 (0.32 – 0.85)  N=15 | 0.27 |
| Epinephrine (µg/kg/min) | 1. (0.0 – 0.08)   N=12 | 1. (0.0 -0.31)   N=4 | 1. (0.0 – 0.08)   N=8 | 0.94 |
| Vasopressin (IE/h) | 0.52 (0.0 – 2) n=15 | 1. (0.0 -2)  n=5 | 1.0 (0 – 2) n=10 | 0.58 |
| Dobutamine (mg/h) | 1. (0.0 – 10) n=13 | 0.0 (0.0 – 10) n=5 | 0.0 (0.0 – 10) n=8 | 0.96 |
| Lactate (mg/dl) | 56 (24 – 95) | 52 (20 – 123) | 60 (27 – 90) | 0.90 |
| **Ventilator Settings and Blood Gas Analysis at VA-ECMO Initiation** | | | | |
| PEEP (mbar) | 13 (12 – 15) | 12 (10 – 13) | 15 (13 – 17) | **0.006** |
| Pmax (mbar) | 30 (26 – 36) | 25 (23 – 32) | 32 (29 – 38) | **0.013** |
| Driving Pressure (mbar) | 15 (14-21) | 14 (13 – 20) | 17 (15 – 22) | **0.03** |
| TV (ml) | 543 (450 – 637) | 538 (450 – 640) | 545 (413 – 642) | 1 |
| TV (ml/kg PDBW) | 7.5 (6.3 – 8.9) | 7.5 (6.5 – 8.9) | 7.5 (6.3 – 8.9) | 0.68 |
| pH | 7.17 (7.1 – 7.21) | 7.15 (7.04 – 7.28) | 7.18 (7.11 – 7.21) | 0.64 |
| P/F Ratio | 71 (59 – 156) | 120 (82 – 218) | 66 (58 – 102) | **0.046** |
| paCO_2_ (mmHg) | 53 (42 – 65) | 45 (35 – 59) | 57 (48 – 69) | 0.07 |
| **Complications during ECMO Therapy** | | | | |
| Acute Kidney Failure | 26 (93) | 9 (82) | 17 (100) | 0.07 |
| Dialysis | 22 (79) | 9 (82) | 13 (76) | 0.74 |
| Intracranial hemorrhage | 5 (18) | 2 (1() | 3 (18) | 0.88 |
| Ischemic Stroke | 1 (4) | 0 (0) | 1 (6) | 0.38 |
| Pulmonary Embolism | 10 (36) | 6 (55) | 4 (24) | 0.10 |

*Table S1: Baseline Characteristics. Continuous variables are shown as median and IQR 25^th^- 75^th^. Categorized variables are shown as number and percentage of group’s size. Statistical difference between groups was calculated using Whitney-Man-U Test for continuous variables and Chi-Square test for categorized variables. Differences in COVID-Treatment and were not calculated due to small case number. *n=12, **n=4, ***n=8*
