# Supplementary material for: Refractory circulatory failure in COVID-19 patients treated with veno-arterial ECMO a retrospective single-center experience
Source: PLoS One. 2024 Apr 1;19(4):e0298342. doi: 10.1371/journal.pone.0298342 (PMC10984404; doi:10.1371/journal.pone.0298342)
Supplement: S2 Table — Summarizes the Patients with Circulatory failure and their specific treatment. * This patient had preexisting reduced LV impairment without deterioration before ECMO. (DOCX) [file pone.0298342.s003.docx]

***Table S2: Patients with Circulatory failure and their specific treatment.***

| Patient | ECMO Mode | Survival | Cause of death | Circulatory failure due to | Vasoplegia | Glucocorticoids | Anakinra | Reconvalescent plasma | Monoclonal AB against COVID-19 | Remdesivir | Tocilizumab | Plasmapheresis | Echocardiography |
| --- | --- | --- | --- | --- | --- | --- | --- | --- | --- | --- | --- | --- | --- |
| 2282 | VA | no | Septic shock | Sepsis | no | yes | no | no | no | no | no | no | Severely reduced systolic Function |
| 2285 | VA | no | Septic shock | Sepsis | yes | no | no | no | no | no | no | no | Unchanged LV Function |
| 2295 | VV >VA | no | Septic shock | Sepsis | yes | yes | yes | yes | no | no | no | no | Unchanged LV Function |
| 2298 | VA | no | Septic shock | Sepsis | yes | yes | no | yes | no | no | yes | yes | Unchanged LV Function |
| 2312 | VA | yes |  | CovLHF | no | yes | yes | yes | no | no | no | yes | Severely reduced systolic Function |
| 2357 | VA | yes |  | CovLHF | no | yes | yes | yes | no | no | no | yes | Severely reduced systolic Function |
| 2427 | VA >VAV | no | Septic shock | Sepsis | yes | yes | no | no | no | yes | no | no | Unchanged LV Function* |
| 2428 | VA | no | Septic shock | Sepsis | no | yes | yes | yes | no | no | no | yes | Severely reduced systolic Function |
| 2448 | VA | no | Septic shock | Sepsis | no | yes | no | yes | no | yes | no | yes | Severely reduced systolic Function |
| 2471 | VV >VA | no | ICH | Sepsis | no | yes | no | yes | no | yes | no | yes | Severely reduced systolic Function |
| 2562 | VA | no | Septic shock | Sepsis | no | yes | no | no | no | no | no | yes | Severely reduced systolic Function |
| 2699 | VA | yes |  | CovLHF | no | yes | no | no | yes | no | no | yes | Severely reduced systolic Function |
| 2766 | VA | yes |  | CovLHF | no | yes | no | no | yes | yes | no | yes | Severely reduced systolic Function |
| 2814 | VA | no | Stroke | Sepsis | No | yes | no | no | no | no | No | no | Severely reduced systolic Function |

*Table S2 summarizes the Patients with Circulatory failure and their specific treatment.* * This patient had preexisting reduced LV impairment without deterioration before ECMO.
