# Supplementary material for: Refractory circulatory failure in COVID-19 patients treated with veno-arterial ECMO a retrospective single-center experience
Source: PLoS One. 2024 Apr 1;19(4):e0298342. doi: 10.1371/journal.pone.0298342 (PMC10984404; doi:10.1371/journal.pone.0298342)
Supplement: S3 Table — Summarizes the causes of death dependent of ECMO indication. ICH: Intracranial hemorrhage. (DOCX) [file pone.0298342.s004.docx]

***Table S3: Causes of death dependent on ECMO indication.***

| **ECMO Indication** | **Cerebral Hypoxia** | **ICH** | **Multiorgan Failure** | **Stroke** | **Septic Shock** |
| --- | --- | --- | --- | --- | --- |
| Circulatory Failure (n=14) | 0 | 1 | 0 | 1 | 8 |
| *Vasoplegia (n=4)* | 0 | 0 | 0 | 0 | 4 |
| CovLHF (n=4) | 0 | 0 | 0 | 0 | 0 |
| Septic ALHF (n=6) | 0 | 1 | 0 | 1 | 4 |
| Cardiac Arrest (n=4) | 1 | 0 | 2 | 0 | 0 |
| Pulmonary embolism (n=5) | 0 | 0 | 0 | 0 | 1 |
| Right heart failure (n=5) | 0 | 0 | 2 | 0 | 1 |

*Table S3 summarizes the causes of death dependent of ECMO indication. ICH: Intracranial hemorrhage.*
