# Supplementary material for: Refractory circulatory failure in COVID-19 patients treated with veno-arterial ECMO a retrospective single-center experience
Source: PLoS One. 2024 Apr 1;19(4):e0298342. doi: 10.1371/journal.pone.0298342 (PMC10984404; doi:10.1371/journal.pone.0298342)
Supplement: S4 Table — Characterizes patients with cardiac arrest. VF: ventricular fibrillation, PEA: pulseless electric activity. NSE: Neuron specific Enolase. (DOCX) [file pone.0298342.s005.docx]

***Table S4 Patients with Cardia arrest.***

| *Patient* | *Initial Rhythm* | *Time Collaps to Cannulation* | *Initial pH* | *Maximal NSE level* | *Initial Lactate* | *Survived* | *Cause of Death* | *Out of Hospital ECPR* |
| --- | --- | --- | --- | --- | --- | --- | --- | --- |
| *2532* | *VF* | *40 min* | *7,11* | *79* | *104 mg/dl* | *No* | *Cerebral Hypoxia* | *yes* |
| *2812* | *VF* | *68 min* | *7,14* | *NA* | *76 mg/dl* | *No* | *MOV* | *yes* |
| *2433* | *VF* | *45 min* | *7,19* | *44* | *123 mg/dl* | *Yes* | *-* | *yes* |
| *2764* | *PEA* | *65 min* | *7,27* | *135* | *87mg/dl* | *No* | *MOV* | *No* |

*Table S4 characterizes patients with cardiac arrest. VF: ventricular fibrillation, PEA: pulsless electric activity. NSE: Neuron specific Enolase.*
