# Supplementary material for: Refractory circulatory failure in COVID-19 patients treated with veno-arterial ECMO a retrospective single-center experience
Source: PLoS One. 2024 Apr 1;19(4):e0298342. doi: 10.1371/journal.pone.0298342 (PMC10984404; doi:10.1371/journal.pone.0298342)
Supplement: S5 Table — Summarizes ECMO configurations and related complications. CFH: cell free Hemoglobin. (DOCX) [file pone.0298342.s006.docx]

***Table S5: ECMO configurations and related complications***

| *ECMO Configuration* | Days from first ECMO configuration to conversion | Acute Kidney Failure | Dialysis | Intracranial hemorrhage | Ischemic Stroke | *Oxygenator Clotting* | *Maximal cell free hemoglobin*  *Median (IQR 25^th^-75^th^) (mg/L)* | *Severe Hemolysis*  *(CFH >500mg/L)* |
| --- | --- | --- | --- | --- | --- | --- | --- | --- |
| *Peripheral VA-ECMO n=17* | *-* | *N=16* | *N=13* | *N=1* | *N=1* | *N=5* | *272 (37 – 612)* | *N=4* |
| *VA to VAV ECMO*  *N=3* | *4* | *N=3* | *N=3* | *N=3* | *N=0* | *N=3* | *380* | *N=0* |
| *VA to VV ECMO*  *N=5* | *4 (3-5)* | *N=4* | *N=4* | *N=0* | *N=0* | *N=4* | *78 (59 – 146)* | *N=0* |
| *VV to VA ECMO*  *N=3* | *6* | *N=3* | *N=2* | *N=1* | *N=0* | *N=0* | *146* | *N=0* |

*Table S5 summarizes ECMO configurations and related complications. CFH: cell free Hemoglobin*
