# Supplementary material for: Refractory circulatory failure in COVID-19 patients treated with veno-arterial ECMO a retrospective single-center experience
Source: PLoS One. 2024 Apr 1;19(4):e0298342. doi: 10.1371/journal.pone.0298342 (PMC10984404; doi:10.1371/journal.pone.0298342)
Supplement: S6 Table — Characterizes patients with right heart failure (RHF). Continuous variables are shown as median and IQR 25th- 75th. Categorized variables are shown as number and percentage of group’s size. (DOCX) [file pone.0298342.s007.docx]

***Table S6: Characteristics of patients with right heart failure***

|  | **All (n=28)** | ***RHF (n=5)*** |
| --- | --- | --- |
| Age | 57.3 (51.4 – 61.8) | *61.9 (57.3 – 65.9)* |
| BMI (kg/m^2^) | 29.4 (26.8 – 33.1) | *26.7 (25.7 – 31.8)* |
| Days from First Symptoms to intubation | 2 (1 – 6) | *5 (1 – 13)* |
| Days from First Symptoms to ECMO | 8 (2 – 19) | *6 (3 – 13)* |
| SOFA | 16 (13 – 17) | *13 (12 – 14)* |
| Murray Score | 3 (2.7 – 3.3) | *2.7 (2.4 – 3.7)* |
| ECMO Duration (days) | 8 (4 – 16) | *8 (3 – 21)* |
| **Pre-Existing Disease** | | |
| Arterial Hypertension | 15 (54) | *3 (60)* |
| Diabetes mellitus | 7 (25) | *0 (0)* |
| Chronic Kidney Insufficiency | 7 (25) | *0 (0)* |
| Immunosuppression | 2 (7) | *0 (0)* |
| Vascular Disease | 3 (11) | *0 (0)* |
| Cardiac Disease | 3 (11) | *0 (0)* |
| Solid Organ Transplantation | 2 (7) | *0 (0)* |
| **Virus Variant** | | |
| Wildtype | 17 (61) | *4 (80)* |
| Alpha-Variant | 4 (14) | *1(20)* |
| Delta-Variant | 5 (18) | *0 (0)* |
| Omikron | 2 (7) | *0 (0)* |
| **Laboratory Testing at ECMO Initiation** | | |
| White blood cells (/nl) | 16.6 (9.3 – 23.9) | *25.3 (11.6 – 43.7)* |
| Lymphocytes (/nl) | 1.3 (0.7 – 1.7) | *1.2 (0.5 – 2.3)* |
| Platelets (/nl) | 241 (102 – 288) | *336 (235 – 382)* |
| Creatinine (mg/dl) | 2.1 (1.1 – 3.0) | *2.6 (1.7 – 4.7)* |
| Blood Urea (mg/dl) | 76 (42 – 115) | *94 (58 – 159)* |
| Bilirubine (mg/dl) | 1.0 (0.5 – 2.4) | *1.4 (1.1 – 3.9)* |
| ASAT (U/L) | 218 (80 – 837) | *1320 (113 – 4179)* |
| ALAT (U/L) | 131 (66 -634) | *1362 (80 – 2654)* |
| LDH (U/L) | 809 (459 – 2839) | *1322 (713 – 4789)* |
| CRP (mg/L) | 187 (60 – 294) | *201 (173 – 262)* |
| Procalcitonin (ng/ml) | 4 (1.1 – 11.3) | *20 (6.5 – 67.3)* |
| IL-6 (pg/ml) | 455 (115 – 1249) | *136 (44 – 1130)* |
| IL-8 (ng/L) | 188 (81 – 560) | *104 (82 – 117)* |
| TNF (pg/ml) | 21.5 (14.0 – 31.2) | *15.5 (11.0 – 30.5)* |
| s-IL2-R (U/ml) | 2013 (869 – 3482) | *2614 (1632 – 3953)* |
| **Ventilator Settings and Blood Gas Analysis at VA-ECMO Initiation** | | |
| PEEP (mbar) | 13 (12 – 15) | *12 (9 – 16)* |
| Pmax (mbar) | 30 (26 – 36) | *27 (25 – 37)* |
| Driving Pressure (mbar) | 15 (14-21) | *17 (13 – 22)* |
| TV (ml) | 543 (450 – 637) | *450 (337 – 600)* |
| TV (ml/kg PDBW) | 7.5 (6.3 – 8.9) | *5.8 (4.3 – 7.7)* |
| pH | 7.17 (7.1 – 7.21) | *7.16 (6.93 – 7.25)* |
| P/F Ratio | 71 (59 – 156) | *96 (66 – 169)* |
| paCO_2_ (mmHg) | 53 (42 – 65) | *55 (38 – 86)* |

*Table S6 characterizes patients with right heart failure (RHF). Continuous variables are shown as median and IQR 25^th^- 75^th^. Categorized variables are shown as number and percentage of group’s size.*
